# Sarcopenia Is Associated With Increased Risks of Rotator Cuff Tendon Diseases Among Community-Dwelling Elders: A Cross-Sectional Quantitative Ultrasound Study

**DOI:** 10.3389/fmed.2021.630009

**Published:** 2021-05-05

**Authors:** Der-Sheng Han, Wei-Ting Wu, Po-Cheng Hsu, Hsiang-Chi Chang, Kuo-Chin Huang, Ke-Vin Chang

**Affiliations:** ^1^Department of Physical Medicine and Rehabilitation, National Taiwan University Hospital, Bei-Hu Branch, Taipei, Taiwan; ^2^Community and Geriatric Medicine Research Center, National Taiwan University Hospital, Bei-Hu Branch, Taipei, Taiwan; ^3^Department of Physical Medicine and Rehabilitation, National Taiwan University College of Medicine, Taipei, Taiwan; ^4^Health Science and Wellness Center, National Taiwan University, Taipei, Taiwan; ^5^Department of Physical Medicine and Rehabilitation, Cheng Hsin General Hospital, Taipei, Taiwan; ^6^Department of Family Medicine, National Taiwan University College of Medicine, Taipei, Taiwan; ^7^Center for Regional Anesthesia and Pain Medicine, Wang-Fang Hospital, Taipei Medical University, Taipei, Taiwan

**Keywords:** ultrasound, shoulder pain, aging, frailty, sarcopenia

## Abstract

**Backgrounds:** Recently, the association between sarcopenia and various musculoskeletal disorders, such as lumbar spine stenosis and fibromyalgia, has been highlighted. However, the relationship between sarcopenia and rotator cuff tendon diseases has rarely been investigated. This study aimed to evaluate whether sarcopenia was associated with shoulder pain and to determine whether rotator cuff tendons differed in echotexture between the sarcopenic and non-sarcopenic populations.

**Methods:** The thickness and echogenicity ratio of the tendon vs. the overlying muscle (ER_TM_) or subcutaneous tissue (ER_TT_) were measured using high-resolution ultrasonography in 56 sarcopenic patients and 56 sex- and age- matched controls. The association between ultrasound measurements of the rotator cuff tendon complex and sarcopenia was investigated using the generalized estimating equation (GEE).

**Results:** The sarcopenic group had an increased prevalence of shoulder pain. Based on the GEE analysis, sarcopenia was significantly associated with an increase in supraspinatus tendon thickness (β coefficient = 0.447, *p* < 0.001) and a decrease in the ER_TM_ for the biceps long head and rotator cuff tendons. A negative trend of association was observed between sarcopenia and ER_TT_ in the supraspinatus tendons (β coefficient = −0.097, *p* = 0.070). Nevertheless, sarcopenia was not associated with an increased risk of rotator cuff tendon tears.

**Conclusions:** Patients with sarcopenia have a higher risk of shoulder pain. A consistent tendinopathic change develops in the supraspinatus tendons in sarcopenic patients. However, sarcopenia is less likely to be associated with serious rotator cuff pathology, such as tendon tears. Prospective cohort studies are warranted to explore the causal relationship between sarcopenia and shoulder disorders.

## Introduction

Sarcopenia, characterized by aging-related gradual loss of muscle performance and function, has a prevalence of ~10% in the population aged > 60 years (1). The causes of sarcopenia are multifactorial, comprising physical inactivity, decline in nutritional intake, degeneration of neuromuscular junctions, and chronic systemic inflammation (2). Currently, an increasing number of potential health consequences of sarcopenia have been uncovered, such as cognitive impairment (3), depression (4), increased mortality (5), and a high risk of falls and mobility limitation (6). The association between sarcopenia and musculoskeletal disorders has been uncovered in the recent years. In patients with lumbar spinal stenosis, sarcopenia was associated with an increased degree of vertebral slippage, more intense lower back pain, and a higher incidence of dyslipidemia and cardiovascular disease (7, 8). A retrospective cohort study also pointed out that grip strength, a widely used parameter for the diagnosis of sarcopenia, was a predictor for the risk of falls in patients following decompression and fusion for lumbar spinal stenosis (9). To date, only a limited number of studies have investigated the relationship between sarcopenia and painful shoulder disorders.

Shoulder pain is a common musculoskeletal complaint, with an annual cumulative incidence of up to 2.4% in adults aged 45–64 years, based on a systematic review (10). Among various types of musculoskeletal pain, painful shoulder disorders have the highest negative impact on the quality of life of middle-aged and elderly people (11). In patients who visited a primary care institute for shoulder problems, the most prevalent pathology arose from the rotator cuff tendons and related structures, such as the subacromial bursa (12). Rotator cuff tendon tears are regarded as the most detrimental type of shoulder tendon disorders and frequently lead to severe disability. In recent years, high-resolution ultrasound has been widely used in the evaluation of rotator cuff tendon disorders (13–15), and its diagnostic accuracy is comparable to that of magnetic resonance imaging (16). In contrast to reports of descriptive findings, quantitative measurements of thickness, and echo intensity provide more objective assessments of tendon texture. Previous studies have demonstrated the capability of quantitative ultrasound in detecting acute echotexture changes in the biceps long head tendon after wheelchair sports (17) and differentiating symptomatic supraspinatus tendinopathy (18). Sarcopenia and painful shoulder syndrome both lead to adverse medical consequences; thus, knowing how they interplay would be helpful for early diagnosis and intervention. Therefore, the present study aimed to evaluate the association between sarcopenia and shoulder pain, and to determine whether the echotexture of rotator cuff tendons changed in sarcopenic patients.

## Methods

### Participant Selection

The participants were community-dwelling elders (age ≥ 65 years) who attended the annual health examination in a community hospital in Taipei, Taiwan. They were excluded if they needed assistance during level walking, had impaired cognitive function, had difficulty complying with instructions and completing the questionnaire, were currently treated for malignancies, had injections/surgeries on either side of the shoulders within 6 months, and had uncontrolled medical conditions (such as severe infection and unstable angina). People with sarcopenia were first enrolled, followed by a sex- and age-matched (±1 year) control group without sarcopenia. The research project was approved by the Institutional Review Board of the National Taiwan University Hospital (IRB No. 201601091RIND). All participants were required to provide written informed consent prior to the commencement of the study.

### Anthropometric Measurement

Body weights and heights of all participants were measured using a standard digital weight and height meter with a minimum scale of 100 g and 1 mm, respectively. Body mass index (BMI) was derived from the weight divided by the height squared (kg/m^2^). Body composition was determined by whole-body dual-energy X-ray absorptiometry (DXA, Stratos dR, DMS Group, France) (19). All participants were required to fast overnight for at least 8 h and wear gowns during examination. The skeletal muscle index (SMI) was derived from the total of four limbs' lean soft tissue (bone-free and fat-free mass, kilogram) divided by the square of the height (m^2^) (19).

### Measurement of Muscle Strength

Grip strength of the participants' dominant hands was measured in the seated position with the elbow flexed at 90° and the forearm supinated. They were asked to forcefully squeeze the isometric dynamometer (Baseline® hydraulic hand dynamometer, Fabrication Enterprises Inc., Irvington, NY, USA) three times with an interval of at least 1 min between each trial. The maximal value was used as the grip strength.

### Diagnosis of Sarcopenia

Sarcopenia was defined as loss of muscle strength and skeletal muscle mass in accordance with the consensus of the European Working Group on Sarcopenia in Older People (20). The cutoff value for low handgrip strength was 30 kg for men and 20 kg for women. An SMI <7.40 kg/m^2^ for men or <5.14 kg/m^2^ for women was considered as low skeletal muscle mass. All included sarcopenic participants were required to fulfill both criteria (21).

### Clinical Evaluation

All participants were required to complete two copies of the Chinese version of the Shoulder Pain and Disability Index (SPADI) tool; one for each shoulder (22). There are 13 items in the tool used to assess shoulder pain and function impairment. Each item has a rating from 0 (no pain or no difficulty) to 10 (worst pain or extreme difficulty). The total score of the SPADI is converted from its pain and functional domains, with a highest value of 100 points. In our study, the presence of shoulder pain was defined as SPADI (total) > 0.

### Ultrasound Scanning Protocol

The examination was performed in accordance with the EURO-MUSCULUS/USPRM shoulder scanning protocol (23). The participant was seated with the arm naturally positioned beside the trunk during the examination of the biceps long head tendon. The transducer was placed across the bicipital groove at the same level as the coracoid process to obtain the short-axis view of the tendon. The transducer was then pivoted 90° to visualize the tendon's long axis. The participant was then asked to externally rotate the arm and the transducer was moved cranially to the coracoid process in the horizontal plane to investigate the subscapularis tendon. Afterwards, the participant was invited to put the hand over the buttock with the elbow pointed backwards, which is the modified Crass position. The transducer was placed lateral to the acromial arch to obtain the long and short axes of the supraspinatus tendon. Finally, the transducer was positioned in the horizontal plane to examine the long axis of the infraspinatus tendon. All the imaging procedures were performed by a physician with 10 years of experience in musculoskeletal ultrasound using a multi-frequency (5–14 MHz) linear transducer (UP 200, BenQ Medical Technology Corporation, Taipei, Taiwan). The physician was unaware of the data of body compositions and physical performance during the ultrasound examination. The scanning depth was set at 40 mm, and the frame rate was set at 300 frames per second. The gain, focus, and dynamic range of the ultrasound machine were kept constant during the examination.

### Ultrasound Diagnostic Protocol

The effusion surrounding the sheath of the long head of the biceps tendon was considered pathological if its thickness was more than 1 mm (24). Subluxation of the long head of the biceps tendon was diagnosed when more than 50% of the tendon's cross-section was outside the bicipital groove. The diagnosis of subdeltoid bursitis was made when the bursa was thicker than 2 mm (24). Intra-tendinous calcification was defined as the presence of hyperechoic plaques with acoustic shadows underneath. Rotator cuff tendon tears were indicated by the existence of visible gaps or total absence of tendon tissue in the subacromial space. As the supraspinatus tendon was large, we further categorized its lesions as full-thickness and partial-thickness tears. A full-thickness tear was identified by an intra-tendinous gap between the subdeltoid bursa and the articular surface of the humeral head (25). In a partial-thickness tear, the gap did not span the entire thickness of the supraspinatus tendon (24).

### Quantitative Ultrasound Measurement

**Figure 1:**
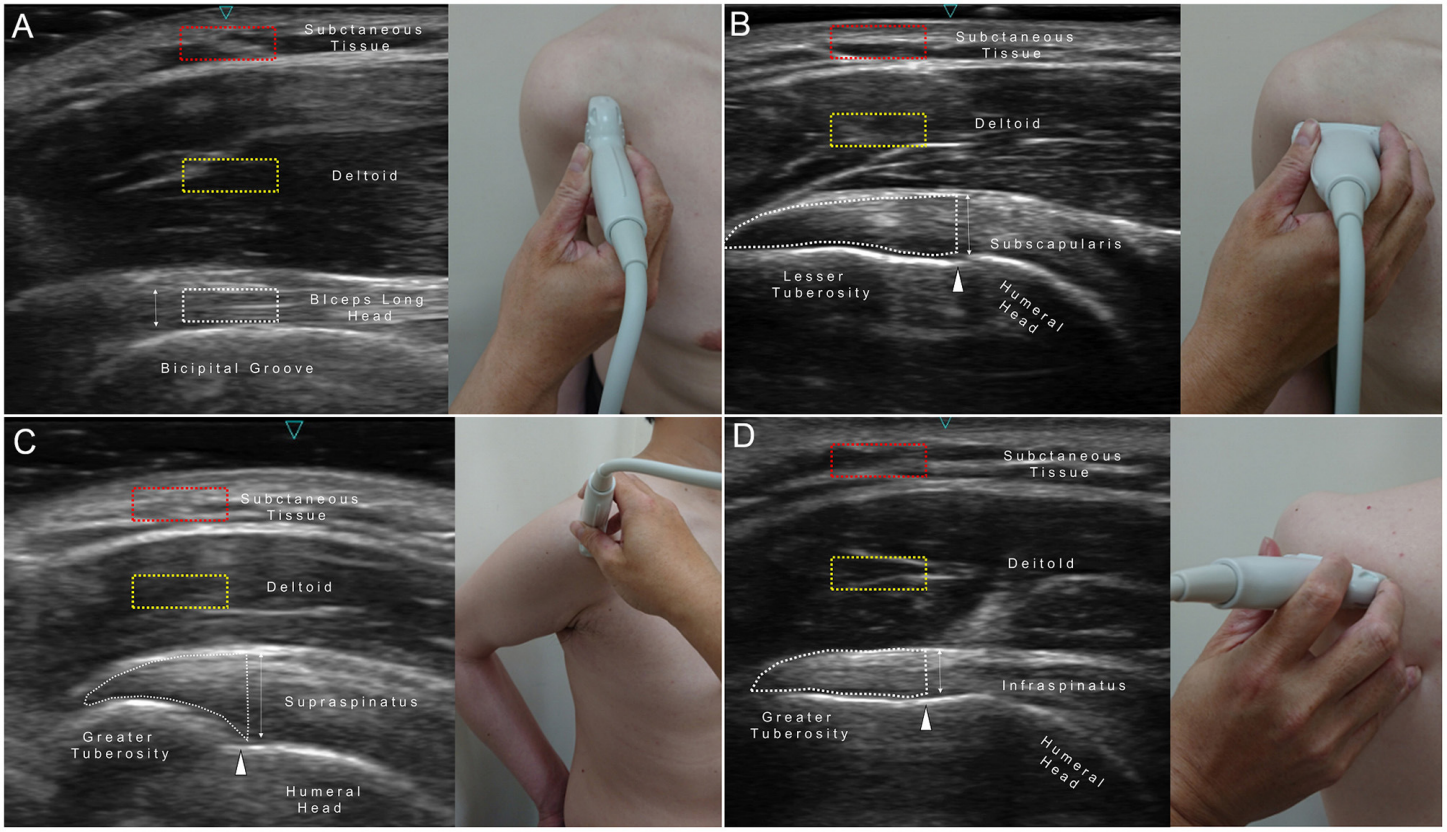
Region of interest (ROI) and site for measuring the echogenicity and thickness of the biceps long head **(A)**, subscapularis **(B)**, supraspinatus **(C)**, and infraspinatus tendons **(D)**. Red dashed squares, ROI for the subcutaneous tissue; yellow dashed squares, ROI for the deltoid muscle; white dashed area, ROI for the target tendon; double arrowed line, tendon thickness; white arrowheads, anatomic neck of the humerus.

Thickness and echogenicity measurements were conducted on the long axis of the tendon using the straight-line tool and histogram function of the image processing software, Image J (Supplementary Figure 1) (15, 24). A line was drawn perpendicular to the most proximal end of the bicipital groove until it reached the superficial border of the tendon to measure the thickness of the biceps long head tendon (Figure 1A). A region of interest (ROI) encircling the cranial 1 cm length of the biceps tendon was selected for echogenicity measurement. Regarding the thickness measurements of the subscapularis (Figure 1B), supraspinatus (Figure 1C), and infraspinatus (Figure 1D) tendons, a vertical line was depicted from the periosteum next to the anatomical neck of the humerus until it reached the superficial edge of the tendon. An ROI encompassing the tendinous area distal to the anatomical neck was marked to obtain echogenicity. A squared ROI (5 × 10 mm) located in the middle of the deltoid muscle over the tendon was employed to calculate the echogenicity ratio of tendon-to-muscle (ER_TM_), denoting the echo intensity of the tendon divided by that of the reference muscle. Likewise, an ROI of the same size was placed at the subcutaneous layer to obtain the echogenicity ratio of tendon-to-tissue (ER_TT_), indicating echo intensity of the tendon divided by that of the subcutaneous tissue. A pilot test was conducted to evaluate the reliability of quantitative ultrasound measurements among five healthy individuals other than the study participants. The intra- and inter-rater reliabilities of the intraclass correlation coefficient (ICC) for measuring tendon thickness were 0.946 and 0.808, respectively. The intra- and inter-rater reliabilities of the ICC for measuring tendon echogenicity were 0.915 and 0.805, respectively. All measurements for the enrolled participants were performed offline by a research assistant who was blinded to their diagnosis.

### Statistical Analysis

The sample size was estimated by identifying a between-group difference of 10 pixels in tendon echogenicity with a standard deviation (SD) of 25 pixels. The alpha level (α) and power (β) were set at 5 and 80%, respectively. The minimum required sample size was 100 shoulders per group.

The mean and SD values were used to report continuous variables, whereas numbers and percentages were used to report categorical variables. Comparisons between normally distributed continuous variables were made using analysis of variance, and the Mann–Whitney *U*-test was used in case the variables were not normally distributed. Categorical variables were compared using the Chi-square test or Fisher's exact test (for sparse data). The association between quantitative ultrasound measurements of shoulder tendons and sarcopenia was investigated using the generalized estimating equation (GEE) (26). The GEE model is widely applied for analyzing longitudinal/clustered data, such as shoulder ultrasound measurements on the right and left sides of the same person. In this model, the participant's identification number served as the clustering variable, while the laterality (right/left side) was imputed as an exchangeable correlation item. Furthermore, Pearson's correlation coefficient (*r*) was applied to measure the correlation of quantitative ultrasound measurements with grip strength and SMI. The analyses were implemented using MedCalc 14.0 (MedCalc Software, Ostend, Belgium) and SPSS 21.0 (IBM SPSS Statistics for Windows, Version 21.0. Armonk, NY: IBM Corp) statistical software, and a *p*-value < 0.05 was considered statistically significant.

## Results

### Demographics and Clinical Assessment

A total of 56 participants with sarcopenia and 56 healthy controls were enrolled. No significant between-group differences were observed in age, sex ratio, height and the prevalence of comorbidities (Supplementary Table 1). Compared with controls, the sarcopenic group had lower body weight, BMI, handgrip strength and SMI. An increased prevalence of shoulder pain and a higher average total SPADI score were observed in the sarcopenic group as compared to the control group (Table 1).

**Table 1:** Descriptive ultrasound findings and clinical evaluation of the study participants.

	**Sarcopenia (+)**	**Sarcopenia (-)**	***p-*value**
	**(shoulder = 112)**	**(shoulder = 112)**	
**Positive ultrasound findings**			
Biceps medial subluxation (number, %)	0 (0.00%)	2 (1.78%)	0.156
	(0.00–0.00%)	(-0.70–4.27%)	
Subscapularis tendon tear (number, %)	4 (3.57%)	2 (1.78%)	0.408
	(0.08–7.06%)	(-0.70–4.27%)	
Subscapularis tendon calcification (number, %)	17 (15.17%)	13 (11.60%)	0.433
	(8.43–21.93%)	(5.58–17.63%)	
Subdeltoid bursitis (number, %)	4 (3.57%)	5 (4.46%)	0.734
	(0.08–7.06%)	(0.58–8.34%)	
Supraspinatus tendon full thickness tear (number, %)	7 (6.25%)	5 (4.46%)	0.553
	(1.69–10.80%)	(0.58–8.34%)	
Supraspinatus tendon partial thickness tear (number, %)	2 (1.78%)	0 (0.00%)	0.156
	(-0.70–4.27%)	(0.00–0.00%)	
Supraspinatus tendon calcification (number, %)	18 (16.07%)	17 (15.17%)	0.854
	(9.16–22.98%)	(8.43–21.93%)	
Infraspinatus tendon tear (number, %)	2 (1.78%)	1 (0.89%)	0.561
	(-0.70–4.27%)	(-0.87–2.66%)	
Infraspinatus tendon calcification (number, %)	6 (5.35%)	6 (5.35%)	1.000
	(1.12–9.59%)	(1.12–9.59%)	
**Clinical evaluation**			
Presence of pain (number, %)	28 (25.00%)	12 (10.71%)	0.005^*^
	(16.86–33.14%)	(4.89–16.53%)	
Pain domain of SPADI (mean ± SD)	1.49 ± 3.15	0.49 ± 1.78	0.004^*^
	(0.90–2.08)	(0.15–0.82)	
Function domain of SPADI (mean ± SD)	0.34 ± 1.12	0.12 ± 0.61	0.068
	(0.13–0.55)	(<0.01–0.24)	
Total score of SPADI (mean ± SD)	0.78 ± 1.66	0.26 ± 1.02	0.005^*^
	(0.47–1.10)	(0.07–0.45)	

Continuous variables are given as mean ± standard deviation and 95% confidence interval. Categorical variables are given as number (percentage) and 95% confidence interval. SPADI, Shoulder Pain and Disability Index. SPADI (total) = [SPADI (pain) *0.5+SPADI (function)*0.8] * (10/13). P-values pertain to between-group comparisons.

* *Indicates p < 0.05*.

### Descriptive and Quantitative Ultrasound Findings

Regarding descriptive ultrasound findings, there were no significant differences in bicep peritendinous effusion, subdeltoid bursitis, calcification, and partial/full-thickness tears of the three rotator cuff tendons (Table 1). In terms of quantitative ultrasound measurements, the sarcopenic group presented with increased thickness and lower ER_TM_ and ER_TT_ of the supraspinatus tendons than those in the control group. Furthermore, the ER_TM_ of the biceps, subscapularis, and infraspinatus tendons was also lower in the sarcopenic group (Table 2). Based on the GEE analysis, sarcopenia was significantly associated with an increase in thickness of the supraspinatus tendon and a decrease in ER_TM_ of the long head of the biceps, subscapularis, and supraspinatus tendons. Sarcopenia was likely to be associated with a decrease in ER_TT_ of the supraspinatus tendon (*p* = 0.07) (Table 3).

**Table 2:** Quantitative measurement of rotator cuff tendon complex in participants with and those without sarcopenia.

	**Sarcopenia (+)**	**Sarcopenia (-)**	***p-*value**
	**(shoulder = 112)**	**(shoulder = 112)**	
**Biceps long head tendon**			
Tendon thickness (mm)	2.34 ± 0.46	2.33 ± 0.42	0.907
	(2.25–2.43)	(2.25–2.41)	
ER_TM_	1.37 ± 0.47	1.88 ± 0.58	<0.001^*^
	(1.29–1.46)	(1.77–1.99)	
ER_TT_	1.83 ± 0.62	1.94 ± 0.65	0.207
	(1.71–1.95)	(1.82–2.06)	
**Subscapularis tendon**			
Tendon thickness (mm)	3.93 ± 0.84	3.74 ± 0.81	0.081
	(3.78–4.09)	(3.59–3.89)	
ER_TM_	1.02 ± 0.46	1.42 ± 0.45	<0.001^*^
	(0.93–1.10)	(1.33–1.50)	
ER_TT_	1.24 ± 0.45	1.30 ± 0.37	0.329
	(1.16–1.33)	(1.23–1.37)	
**Supraspinatus tendon**			
Tendon thickness (mm)	5.49 ± 1.15	4.83 ± 1.06	<0.001^*^
	(5.27–5.70)	(4.63–5.03)	
ERTM	0.87 ± 0.32	1.30 ± 0.52	<0.001^*^
	(0.81–0.93)	(1.20–1.40)	
ERTT	1.08 ± 0.31	1.18 ± 0.38	0.042^*^
	(1.02–1.14)	(1.11–1.25)	
**Infraspinatus tendon**			
Tendon thickness (mm)	3.86 ± 1.24	3.65 ± 1.01	0.163
	(3.63–4.09)	(3.46–3.84)	
ERTM	0.90 ± 0.34	1.20 ± 0.35	<0.001^*^
	(0.84–0.97)	(1.13–1.27)	
ERTT	1.28 ± 0.42	1.40 ± 0.57	0.096
	(1.20–1.36)	(1.29–1.50)	

*Values are given as mean ± standard deviation and 95% confidence interval. ER_TM_, echogenicity ratio of the tendon vs. the overlying deltoid muscle; ER_TT_, echogenicity ratio of the tendon vs. the overlying subcutaneous tissue. P-values pertain to between-group comparisons*.

* *Indicates p < 0.05*.

**Table 3:** Association of sarcopenia and demographics with quantitative ultrasound measurements of rotator cuff tendon complex.

	**Biceps long head tendon**			**Subscapularis tendon**			**Supraspinatus tendon**			**Infraspinatus tendon**		
	**Thickness**	**ER_**TM**_**	**ER_**TT**_**	**Thickness**	**ER_**TM**_**	**ER_**TT**_**	**Thickness**	**ER_**TM**_**	**ER_**TT**_**	**Thickness**	**ER_**TM**_**	**ER_**TT**_**
Sarcopenia	−0.022	−0.599	−0.065	0.173	−0.442	−0.051	0.703	−0.447	−0.097	0.144	−0.277	−0.050
	(−0.16 to 0.11)	(−0.76 to −0.43)	(−0.30 to 0.17)	(−0.10 to 0.45)	(−0.57 to −0.30)	(−0.18 to 0.08)	(0.32 to 1.08)	(−0.57 to −0.31)	(−0.20 to 0.008)	(−0.23 to 0.51)	(−0.38 to −0.17)	(−0.21 to 0.11)
	*p* = 0.757	*p* <0.001^*^	*p* = 0.587	*p* = 0.224	*p* <0.001^*^	*p* = 0.469	*p* <0.001^*^	*p* <0.001^*^	*p* = 0.070^*^	*p* = 0.451	*p* <0.001^*^	*p* = 0.548
Age (years)	0.005	0.004	0.001	0.009	−0.005	0.002	−0.029	−0.003	−0.001	−0.013	0.005	0.011
	(−0.006 to 0.01)	(−0.01 to 0.02)	(−0.01 to 0.02)	(−0.01 to 0.03)	(−0.01 to 0.008)	(−0.009 to 0.01)	(−0.05 to 0.00)	(−0.01 to 0.009)	(−0.01 to 0.008)	(−0.03 to 0.01)	(−0.005 to 0.01)	(−0.004 to 0.02)
	*p* = 0.350	*p* = 0.607	*p* = 0.875	*p* = 0.425	*p* = 0.490	*p* = 0.743	*p* = 0.052	*p* = 0.660	*p* = 0.780	*p* = 0.327	*p* = 0.305	*p* = 0.166
Female gender	−0.182	−0.350	0.157	−0.092	−0.194	0.106	0.071	−0.341	−0.160	0.072	0.010	0.226
	(−0.37 to 0.07)	(−0.61 to −0.09)	(−0.16 to 0.48)	(−0.48 to 0.30)	(−0.43 to 0.04)	(−0.10 to 0.32)	(−0.42 to 0.57)	(−0.55 to −0.12)	(−0.30 to −0.01)	(−0.35 to 0.49)	(−0.12 to 0.14)	(−0.04 to 0.49)
	*p* = 0.059	*p* = 0.008	*p* = 0.345	*p* = 0.649	*p* = 0.112	*p* = 0.334	*p* = 0.779	*p* = 0.002^*^	*p* = 0.027^*^	*p* = 0.740	*p* = 0.887	*p* = 0.105
Height (cm)	−0.003	−0.007	0.002	−0.004	−0.003	0.000	0.001	0.001	0.002	−0.002	0.001	0.003
	(−0.008 to 0.003)	(−0.01 to 0.001)	(−0.009 to 0.01)	(−0.01 to 0.008)	(−0.009 to 0.004)	(−0.006 to 0.007)	(−0.01 to 0.01)	(−0.005 to 0.007)	(−0.002 to 0.006)	(−0.01 to 0.01)	(−0.003 to 0.005)	(−0.006 to 0.01)
	*p* = 0.382	*p* = 0.090	*p* = 0.671	*p* = 0.478	*p* = 0.446	*p* = 0.934	*p* = 0.850	*p* = 0.760	*p* = 0.359	*p* = 0.691	*p* = 0.534	*p* = 0.558
Weight (kg)	−0.003	−0.009	0.004	−0.001	−0.004	0.000	0.005	−0.001	0.000	−0.007	0.002	0.006
	(−0.007 to 0.002)	(−0.01 to −0.001)	(−0.006 to 0.01)	(−0.01 to 0.01)	(−0.01 to 0.003)	(−0.006 to 0.007)	(−0.01 to 0.02)	(−0.008 to 0.005)	(−0.005 to 0.004)	(−0.01 to 0.005)	(−0.002 to 0.005)	(−0.002 to 0.01)
	*p* = 0.270	*p* = 0.026^*^	*p* = 0.411	*p* = 0.827	*p* = 0.233	*p* = 0.918	*p* = 0.498	*p* = 0.648	*p* = 0.812	*p* = 0.247	*p* = 0.420	*p* = 0.130
Left side	0.002	0.142	0.055	0.162	0.020	0.085	0.022	−0.046	−0.059	−0.049	0.077	0.063
	(−0.10 to 0.10)	(0.03 to 0.25)	(−0.06 to 0.17)	(−0.003 to 0.32)	(−0.07 to 0.11)	(−0.004 to 0.17)	(−0.21 to 0.26)	(−0.13 to 0.04)	(−0.13 to 0.01)	(−0.29 to 0.19)	(−0.005 to 0.15)	(−0.02 to 0.15)
	*p* = 0.966	*p* = 0.011^*^	*p* = 0.363	*p* = 0.054	*p* = 0.685	*p* = 0.062	*p* = 0.855	*p* = 0.307	*p* = 0.130	*p* = 0.696	*p* = 0.067	*p* = 0.180

*ER_TM_, echogenicity ratio of the tendon vs. the overlying deltoid muscle; ER_TT_, echogenicity ratio of the tendon vs. the overlying subcutaneous tissue*.

* *indicates p < 0.05*.

### Correlation of Ultrasound Measurements With Grip Strength and SMI

**Figure 2:**
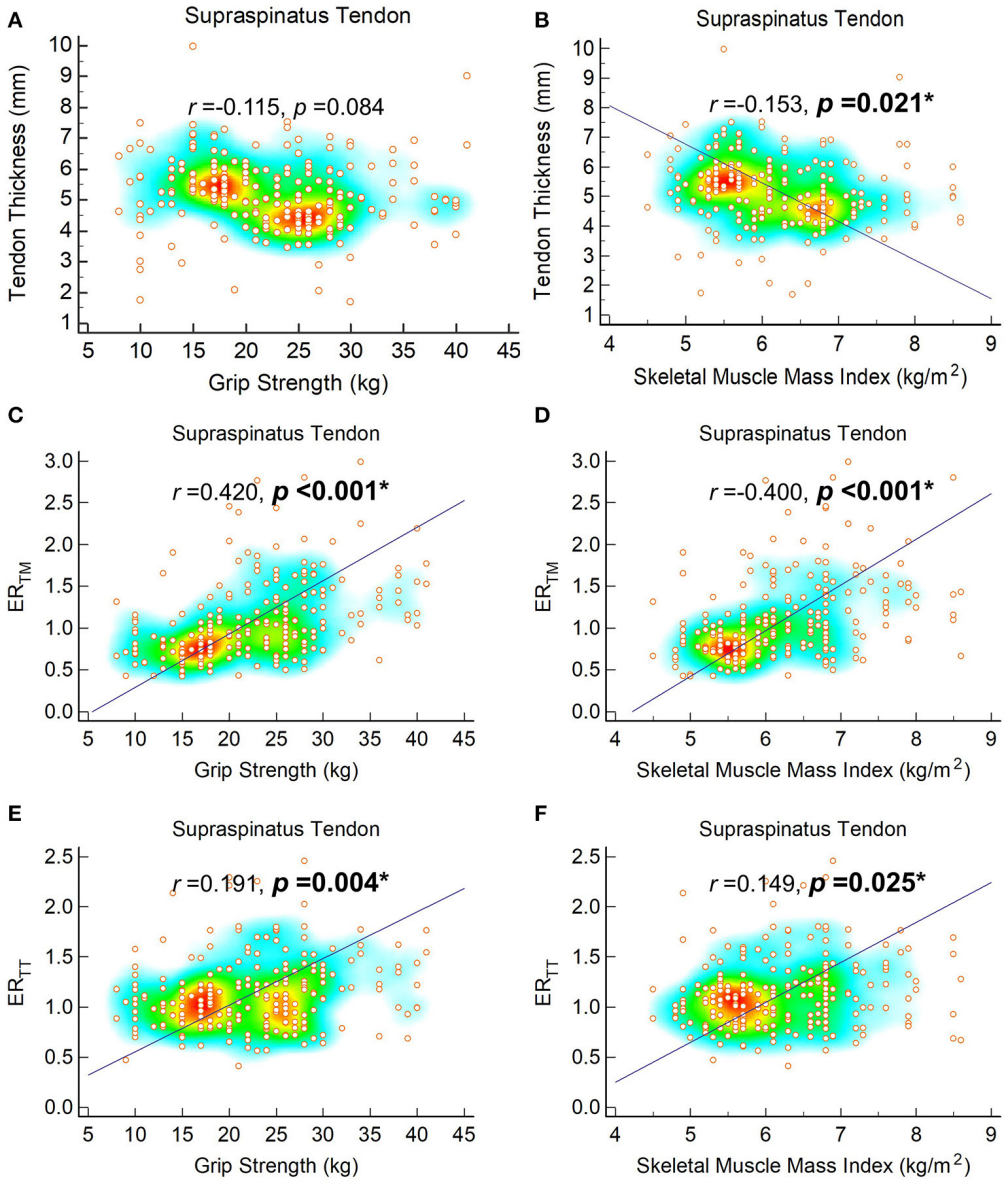
Correlation of tendon thickness with grip strength **(A)** and skeletal muscle mass index **(B)**, ER_TM_ with grip strength **(C)** and skeletal muscle mass index **(D)** and ER_TT_ with grip strength **(E)** and skeletal muscle mass index **(F)** of the supraspinatus tendons. The regression line is plotted on when *p*-value is < 0.05. The heat map with background color coding suggests clusters of observations. ER_TM_, echogenicity ratio of the tendon vs. the overlying deltoid muscle; ER_TT_, echogenicity ratio of the tendon vs. the overlying subcutaneous tissue.

The correlation analyses indicated significant positive relationships between grip strength and ER_TM_ of the four tendons. Likewise, SMI was positively correlated with ER_TT_ of the supraspinatus tendons, and ER_TM_ measurements of the four tendons (Figure 2, Supplementary Figures 2–4). Nevertheless, SMI was negatively correlated with the thickness of the supraspinatus tendons. A trend of negative correlation (*p* = 0.084) was observed between grip strength and thickness of the supraspinatus tendon (Figure 2).

### Echogenicity of Target Tendons, Overlying Muscles, and Subcutaneous Tissue

Furthermore, between-group comparisons were conducted for echogenicity of the four tendons as well as adjacent deltoid muscles and overlying subcutaneous tissues, used for calculating ER_TM_ and ER_TT_. Although mean values of tendon echogenicity appeared lower in the sarcopenic group, none of the comparisons were statistically significant. However, the echogenicity of the deltoid muscles on top of the four tendons was significantly higher in the sarcopenic group. No between-group differences were observed in the echogenicity of the subcutaneous tissues (Supplementary Table 2).

## Discussion

In this study, based on data from community-dwelling elders, we discovered that those with sarcopenia had a higher prevalence of shoulder pain. Sarcopenia was significantly associated with an increase in supraspinatus tendon thickness and a decrease in ER_TM_ for the biceps long head and rotator cuff tendons. A negative trend of association between sarcopenia and ER_TT_ of supraspinatus tendons was observed. Nevertheless, sarcopenia was not associated with an increased risk of rotator cuff tendon tears.

The relationship between sarcopenia and musculoskeletal pain has been uncovered in recent years. In 2015, Koca et al. studied body composition and physical performance of patients with fibromyalgia syndrome and discovered that the fibromyalgia group had significantly lower grip strength than healthy controls (27). In 2019, Sit et al. conducted a survey among elderly patients (age ≥ 60 years) from primary care clinics and found that sarcopenia, defined by a score ≥ 4 on the SARC-F (Strength, Assistance with walking, Rise from a chair, Climb stairs and Falls) questionnaire, was associated with chronic musculoskeletal pain (28). Their study also pointed out that patients with sarcopenia were more likely to present with pain in multiple sites. In 2020, Imagama et al. identified an independent association between sarcopenia and neuropathic pain among community-dwelling middle-aged and elderly volunteers. Several possible mechanisms have been proposed for interpreting the link between sarcopenia and musculoskeletal pain. First, pain limits the activities of the affected limbs, which further leads to disuse muscle atrophy (29). Second, weakened muscles in sarcopenic participants fail to provide adequate stability of the joints, which elicits repeated injury of juxta-articular structures (28). Third, sarcopenia is similar to a subclinical state of inflammation, wherein increased levels of circulating pro-inflammatory cytokines, such as IL-6 and TNFα, might potentiate pain sensitivity of the musculoskeletal system (30).

**Figure 3:**
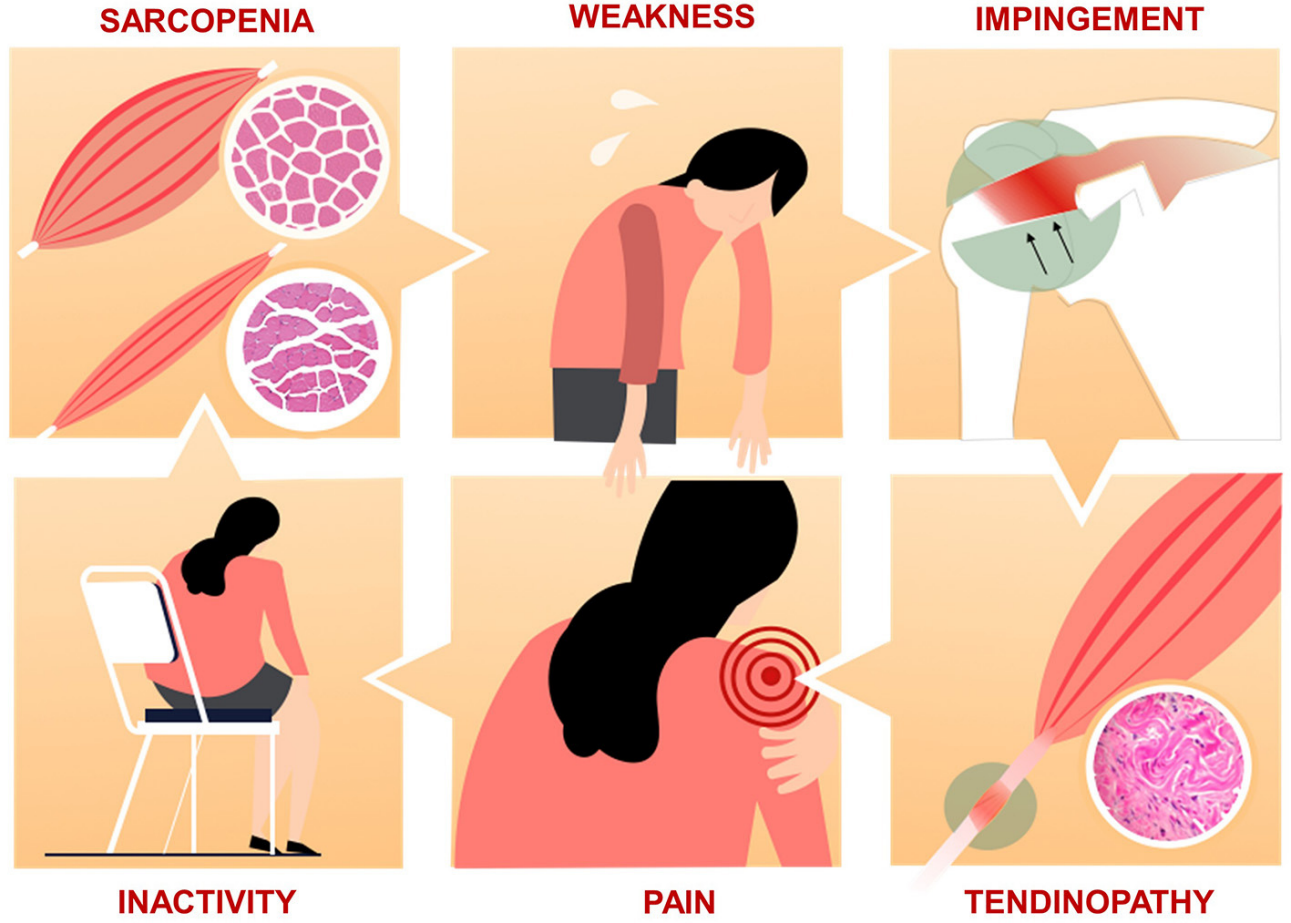
A plausible diagram illustrating how sarcopenia interacts with rotator cuff tendon diseases.

Our study found a higher prevalence of shoulder pain and increased impairment of shoulder function in the sarcopenic group than in healthy controls. However, the number of shoulders with discomfort only accounted for appropriately 25% of the sarcopenic group. Since there were no symptoms in a majority of our participants, pain was less likely to be the main cause of sarcopenia. In contrast, as sarcopenia is characterized by a decline in muscle mass and function (21), our study has identified its effect on muscles over the shoulder girdle. The echogenicity of the deltoid muscle over the four target tendons appeared higher in the sarcopenic group, implying fatty infiltration and atrophic changes (31). Therefore, we support the theory that sarcopenia resulted in weakness and incoordination of the shoulder girdle muscles, and subsequent tendon injury due to joint instability. Furthermore, in patients who already have shoulder pain, their symptoms might lead to physical inactivity and further worsen the status of sarcopenia. A vicious cycle of sarcopenia- pain-inactivity-sarcopenia is thus formed (Figure 3). However, as this is a cross-sectional study, the aforementioned speculation might be just a statistical correlation. Patients with sarcopenia are likely to be comorbid with many chronic diseases, like diabetes mellitus (32), which has been proven to affect the quality of rotator cuff tendons (33). We believed a longitudinal study would be needed for clarifying the biological correlation between sarcopenia and shoulder pathologies.

Increased thickness and decreased echogenicity are the hallmarks of tendinopathy in ultrasound imaging, reflecting myxoid degeneration and disorganization of the collagen bundles (34). Among all tendons in the rotator cuff complex, our findings revealed that the supraspinatus tendons in the sarcopenic group consistently presented with degenerative changes, comprising an increase in tendon thickness and a decline in ER_TM_ and ER_TT_. Our correlation analyses also implied that worsening of the quantitative parameters of the supraspinatus tendons was mostly correlated with a decline in grip strength and SMI. The supraspinatus muscle plays an important role in shoulder abduction and external rotation, and its tendon has been shown to be more frequently affected than other rotator cuff tendons (35). It also helps to depress the humeral head during overhead activity to prevent subacromial impingement (35). In patients with sarcopenia, we speculated that supraspinatus muscles might be affected/weakened like deltoid muscles, which led to a narrower subacromial space upon arm elevation and subsequent tendon injury due to impingement.

The ER_TM_ is a common ultrasound parameter to evaluate tendon quality (18, 25). While isolating a deeper structure, the target appears more hypoechoic due to absorption of ultrasound energy (36). Using echogenicity of the overlying muscles as the reference, the ER_TM_ can partly counteract the influences of signal gain at various depths, which is considered better than using tendon echogenicity alone for comparisons among different subjects. Furthermore, compared with the subcutaneous tissue, the overlying muscle is less compressible and serves as a superior reference target for calculating the echogenicity ratio. However, when applying the ER_TM_ for shoulder tendons on participants with sarcopenia, the values could have been underestimated, as the echogenicity of the deltoid muscles was increased (Supplementary Table 2). Therefore, we also validated the tendon echotexture by employing ER_TT_ and found that the biceps long head, subscapularis, and supraspinatus tendons had decreased ER_TM_, but not ER_TT_. As the thickness of the biceps long head, subscapularis, and supraspinatus tendons did not differ among participants with and without sarcopenia, there was no robust evidence to prove the decline in their tendon echotexture.

Our results showed no significant differences in the prevalence of rotator cuff tendon tears between participants with and without sarcopenia. In 2016, Chung et al. compared the sarcopenia index between patients with surgically proven rotator cuff tendon tears and healthy participants (37). They found that the grip strength and SMI tended to be lower in the group with torn tendons than in the age- and sex-matched controls. In addition, patients with large tears had an inferior sarcopenia index compared to those with small tears. Rotator cuff syndrome is a spectrum of diseases, ranging from tendinopathy to tendon tears, and the latter pathology commonly causes severe pain and disability (38). Unlike Chung et al., who enrolled patients with rotator cuff tendon tears needing surgical intervention, our participants were mostly asymptomatic. Therefore, although certain pathologic changes could be observed in the supraspinatus tendons in the sarcopenic group, the majority of their symptoms remained mild without progression to tendon ruptures.

Several limitations of our study should be noted. First, we used a cross-sectional design, and the causal relationship between sarcopenia and rotator cuff tendon disorders could not be clarified through our analysis. A cohort study with longitudinal follow up of body composition, physical performance and shoulder ultrasonography would be helpful in validation of the biological association between sarcopenia and shoulder pathologies. Second, we did not measure strength of the muscles in the shoulder girdle and we were not aware if weakness could be identified in rotator cuff muscles of participants with sarcopenia. Isokinetic testing of shoulder abductors/adductors, flexors/extensors, and external/internal rotators should be incorporated in future studies. Third, the case number in the present study was relatively small. The differences in echotexture of the rotator cuff tendons might not be limited to the supraspinatus tendons if the statistical power can be improved with an increase in the participant number. Therefore, a large-scale study is still needed for verifying our preliminary observation.

## Conclusion

Patients with sarcopenia have a high risk of shoulder pain. A consistent pathological change develops in the supraspinatus tendons of sarcopenic patients. Nevertheless, sarcopenia is less likely to be associated with serious rotator cuff pathology, such as tendon tears. Prospective cohort studies are warranted to assess the relationship between sarcopenia and various shoulder disorders and the influence of different kinds of comorbidities on rotator cuff tendon echotextures.

## Data Availability Statement

The original contributions presented in the study are included in the article/Supplementary Material, further inquiries can be directed to the corresponding author/s.

## Ethics Statement

The studies involving human participants were reviewed and approved by Research Ethics Committee of National Taiwan University Hospital (IRB No. 201601091RIND). The patients/participants provided their written informed consent to participate in this study.

## Author Contributions

K-VC wrote the manuscript. W-TW and P-CH analyzed the data. D-SH interpreted the results. K-VC, W-TW, and K-CH enrolled eligible patients. H-CC edited and drew the pictures. All authors participated in the design of the study, reviewed the manuscript, contributed to the discussion, and read and approved the final manuscript.

## Conflict of Interest

The authors declare that the research was conducted in the absence of any commercial or financial relationships that could be construed as a potential conflict of interest.

## Supplementary Material

Supplementary Figure 1 The line tool **(A)** and histogram **(B)** for measuring thickness and echogenicity.

Supplementary Figure 2 Correlation of tendon thickness with grip strength **(A)** and skeletal muscle mass index **(B)**, ER_TM_ with grip strength **(C)** and skeletal muscle mass index **(D)** and ER_TT_ with grip strength **(E)** and skeletal muscle mass index **(F)** of the biceps long head tendons. The regression line is plotted on when *p*-value is < 0.05. The heat map with background color coding suggests clusters of observations. ER_TM_, echogenicity ratio of the tendon vs. the overlying deltoid muscle; ER_TT_, echogenicity ratio of the tendon vs. the overlying subcutaneous tissue.

Supplementary Figure 3 Correlation of tendon thickness with grip strength **(A)** and skeletal muscle mass index **(B)**, ER_TM_ with grip strength **(C)** and skeletal muscle mass index **(D)** and ER_TT_ with grip strength **(E)** and skeletal muscle mass index **(F)** of the subscapularis tendons. The regression line is plotted on when *p* value is < 0.05. The heat map with background color coding suggests clusters of observations. ER_TM_, echogenicity ratio of the tendon vs. the overlying deltoid muscle; ER_TT_, echogenicity ratio of the tendon vs. the overlying subcutaneous tissue.

Supplementary Figure 4 Correlation of tendon thickness with grip strength **(A)** and skeletal muscle mass index **(B)**, ER_TM_ with grip strength **(C)** and skeletal muscle mass index **(D)** and ER_TT_ with grip strength **(E)** and skeletal muscle mass index **(F)** of the infraspinatus tendons. The regression line is plotted on when *p*-value is < 0.05. The heat map with background color coding suggests clusters of observations. ER_TM_, echogenicity ratio of the tendon vs. the overlying deltoid muscle; ER_TT_, echogenicity ratio of the tendon vs. the overlying subcutaneous tissue.

## Abbreviations

ER_TM_: echogenicity ratio of the tendon vs. the overlying muscle
ER_TT_: echogenicity ratio of the tendon vs. the subcutaneous tissue
GEE: generalized estimating equation
BMI: body mass index
SMI: skeletal muscle index
SPADI: shoulder pain and disability index
ROI: region of interest
ICC: intraclass correlation coefficient
SD: standard deviation.
