# Supplementary figures and images for: Sarcopenia Is Associated With Increased Risks of Rotator Cuff Tendon Diseases Among Community-Dwelling Elders: A Cross-Sectional Quantitative Ultrasound Study
Source: Front Med (Lausanne). 2021 May 5;8:630009. doi: 10.3389/fmed.2021.630009 (PMC8131871; doi:10.3389/fmed.2021.630009)

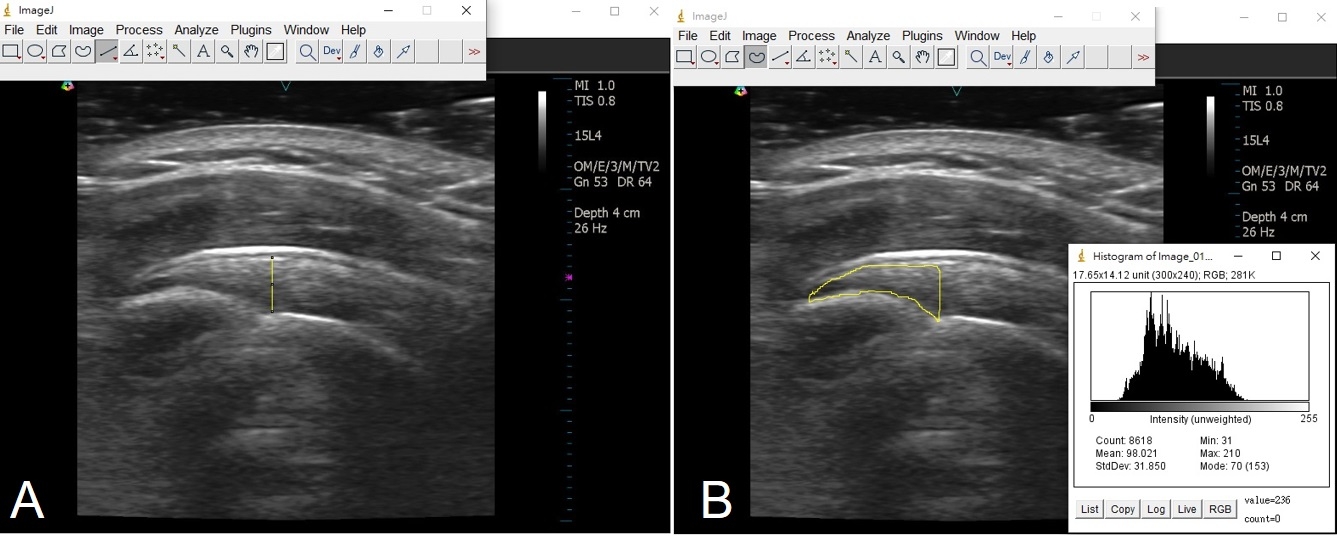

Supplement: Supplementary Figure 1 — The line tool (A) and histogram (B) for measuring thickness and echogenicity. [file Image_1.JPEG]

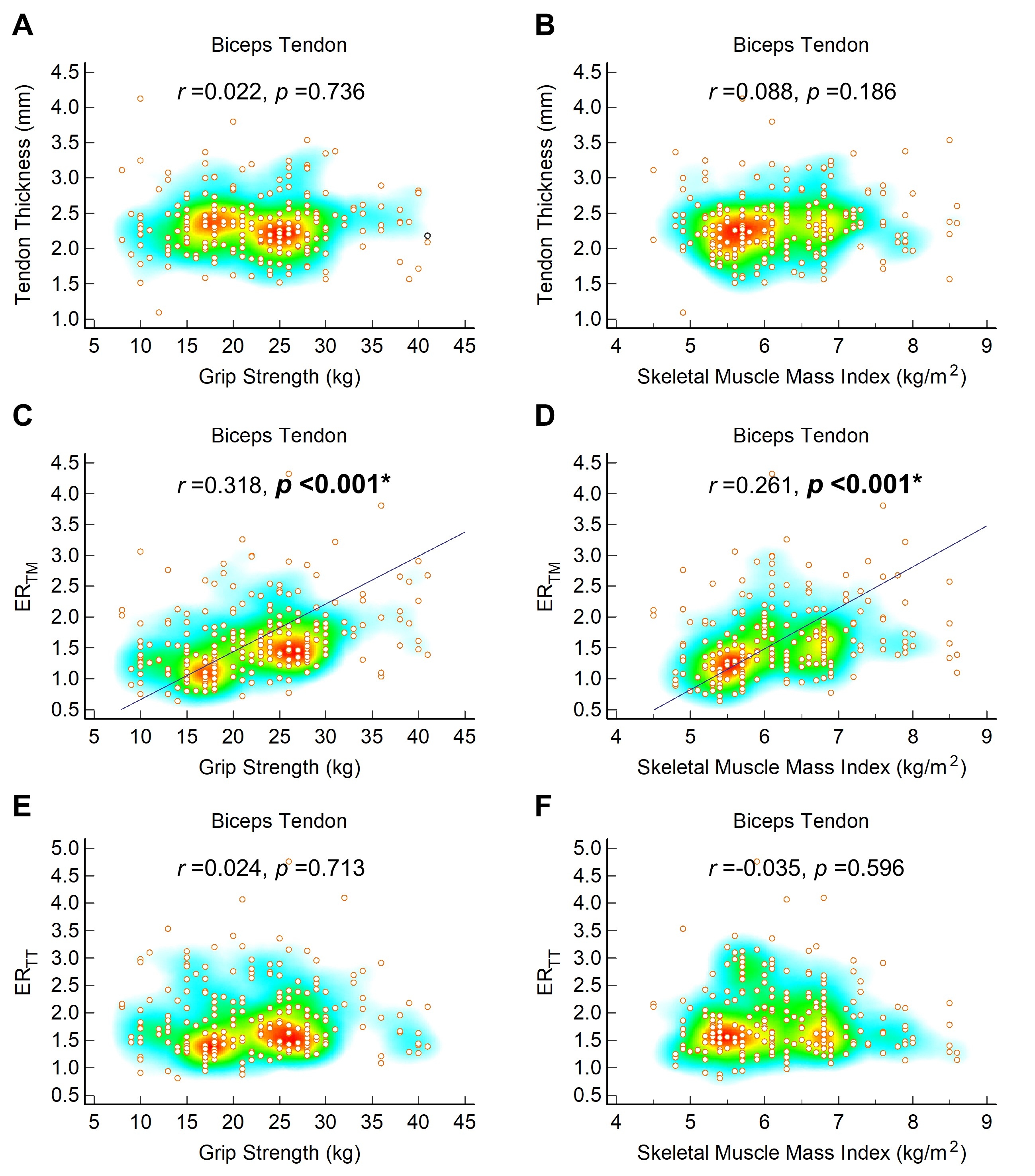

Supplement: Supplementary Figure 2 — Correlation of tendon thickness with grip strength (A) and skeletal muscle mass index (B), ERTM with grip strength (C) and skeletal muscle mass index (D) and ERTT with grip strength (E) and skeletal muscle mass index (F) of the biceps long head tendons. The regression line is plotted on when p-value is < 0.05. The heat map with background color coding suggests clusters of observations. ERTM, echogenicity ratio of the tendon vs. the overlying deltoid muscle; ERTT, echogenicity ratio of the tendon vs. the overlying subcutaneous tissue. [file Image_2.JPEG]

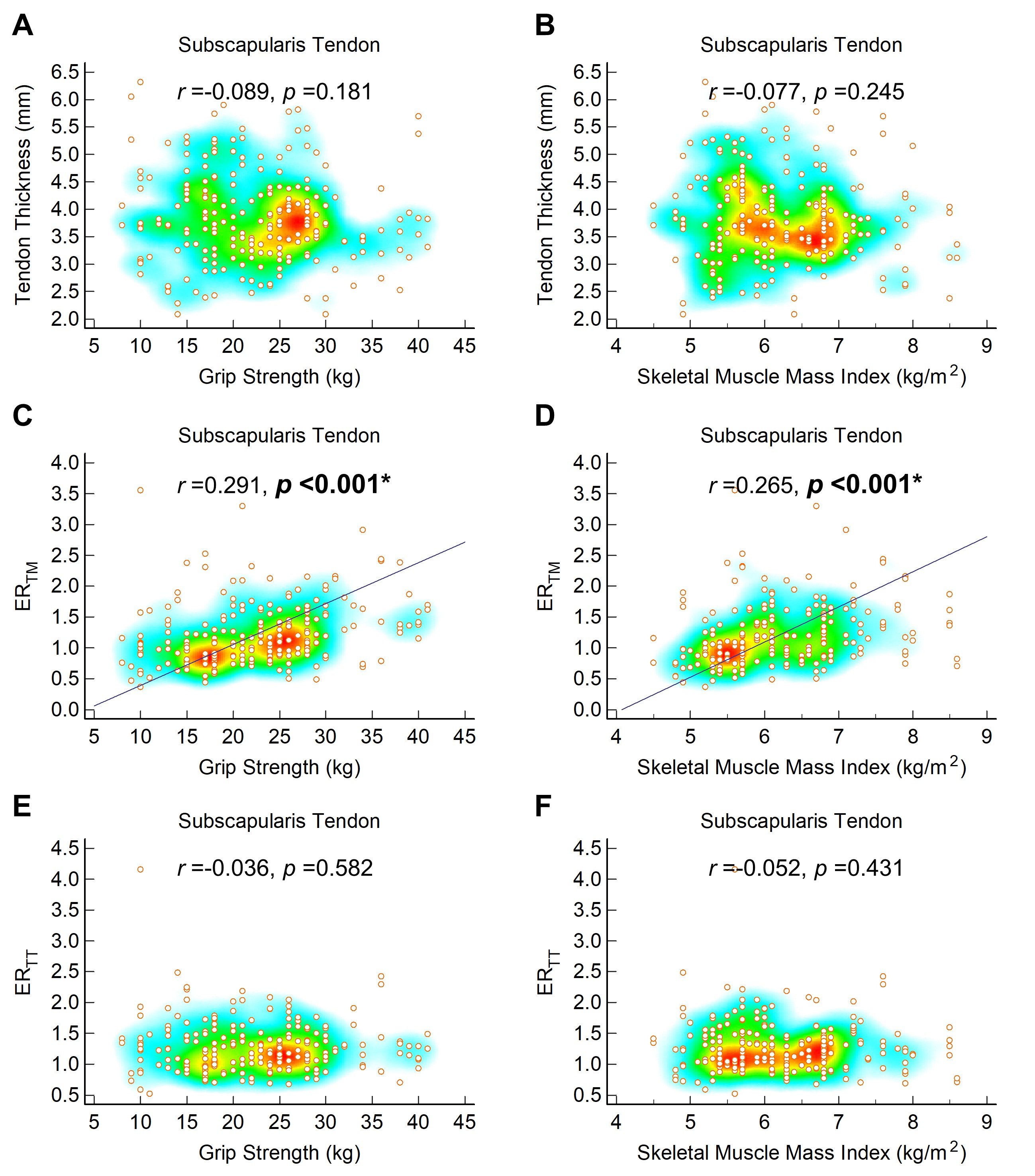

Supplement: Supplementary Figure 3 — Correlation of tendon thickness with grip strength (A) and skeletal muscle mass index (B), ERTM with grip strength (C) and skeletal muscle mass index (D) and ERTT with grip strength (E) and skeletal muscle mass index (F) of the subscapularis tendons. The regression line is plotted on when p value is < 0.05. The heat map with background color coding suggests clusters of observations. ERTM, echogenicity ratio of the tendon vs. the overlying deltoid muscle; ERTT, echogenicity ratio of the tendon vs. the overlying subcutaneous tissue. [file Image_3.JPEG]

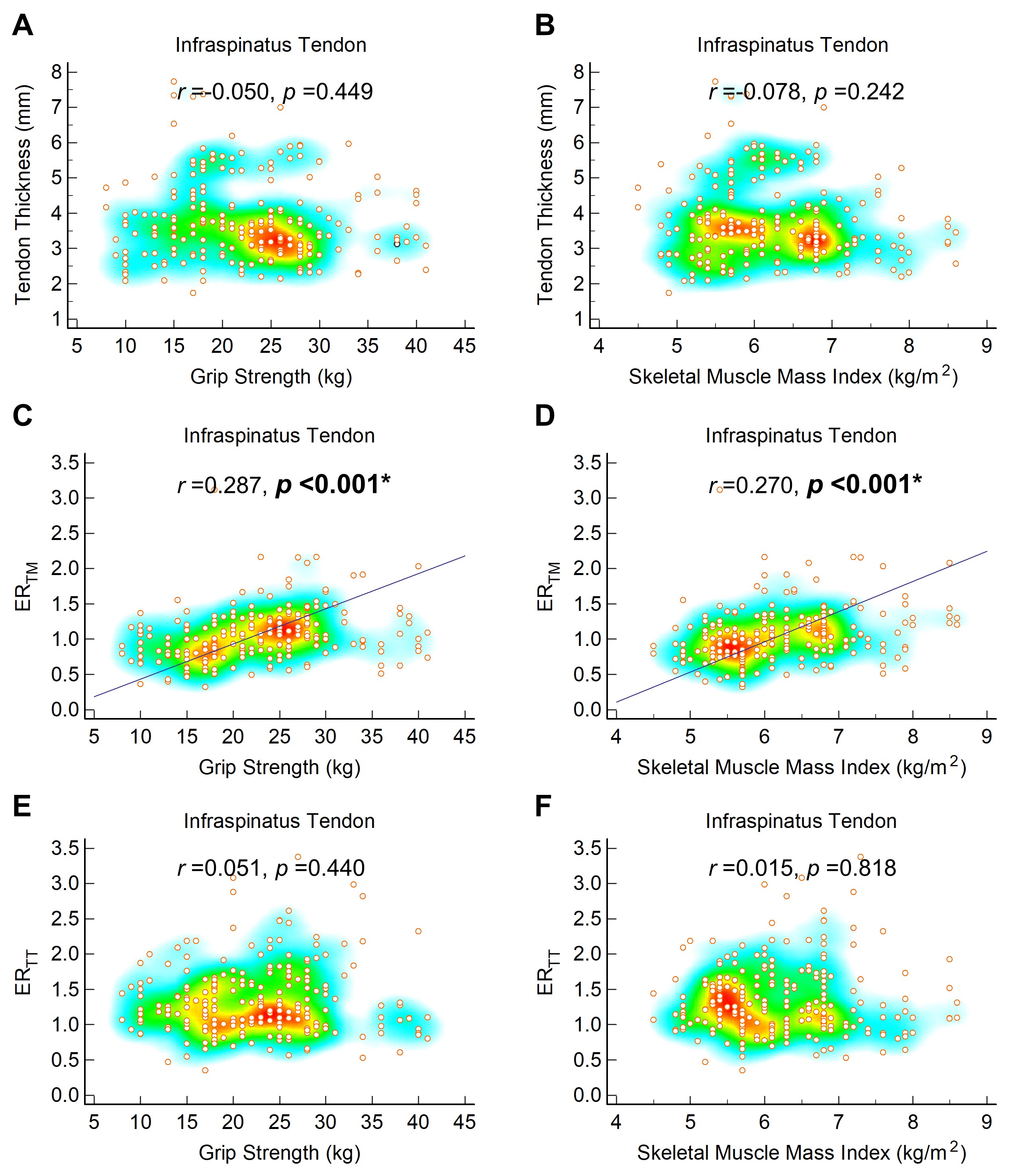

Supplement: Supplementary Figure 4 — Correlation of tendon thickness with grip strength (A) and skeletal muscle mass index (B), ERTM with grip strength (C) and skeletal muscle mass index (D) and ERTT with grip strength (E) and skeletal muscle mass index (F) of the infraspinatus tendons. The regression line is plotted on when p-value is < 0.05. The heat map with background color coding suggests clusters of observations. ERTM, echogenicity ratio of the tendon vs. the overlying deltoid muscle; ERTT, echogenicity ratio of the tendon vs. the overlying subcutaneous tissue. [file Image_4.JPEG]
