# Supplementary material for: Sarcopenia Is Associated With Increased Risks of Rotator Cuff Tendon Diseases Among Community-Dwelling Elders: A Cross-Sectional Quantitative Ultrasound Study
Source: Front Med (Lausanne). 2021 May 5;8:630009. doi: 10.3389/fmed.2021.630009 (PMC8131871; doi:10.3389/fmed.2021.630009)
Supplement: Supplementary file 5 [file Table_1.docx]

**Supplementary Table 1.** Demographics, physical performance and body composition of the study participants

|  | **Sarcopenia (+)**  **(N=56)** | **Sarcopenia (-)**  **(N=56)** | ***p* value** |
| --- | --- | --- | --- |
| Demographics and anthropometrics measurements | | | |
| Age (year) | 75.12 ± 5.91  (73.54 to 76.70) | 75.16 ± 5.54  (73.67 to 76.64) | 0.974 |
| Female gender (number, %) | 43 (76.78%)  (65.38% to 88.19%) | 43 (76.78%)  (65.38% to 88.19%) | 1.000 |
| Height (cm) | 150.97 ± 21.40  (145.24 to 156.70) | 152.86 ± 18.2  (147.98 to 157.73) | 0.616 |
| Weight (kg) | 56.65 ± 19.51  (51.42 to 61.88) | 65.89 ± 19.81  (60.58 to 71.19) | 0.014* |
| Body mass index (kg/m^2^) | 22.39 ± 3.64  (21.41 to 23.37) | 25.72 ± 2.99  (24.91 to 26.52) | <0.001* |
| Physical performance |  |  |  |
| Handgrip strength (kg) | 17.50 ± 5.84  (15.93 to 19.06) | 27.48 ± 5.65  (25.96 to 28.99) | <0.001* |
| Body Composition | | | |
| Skeletal muscle mass index (kg/ m^2^) | 5.61 ± 0.56  (5.46 to 5.76) | 6.82 ± 0.73  (6.62 to 7.01) | <0.001* |

Values are given as mean ± standard deviation and 95% confidence interval. *P* values pertain to between-group comparisons. * indicates *p* <0.05.
