# Supplementary material for: Sarcopenia Is Associated With Increased Risks of Rotator Cuff Tendon Diseases Among Community-Dwelling Elders: A Cross-Sectional Quantitative Ultrasound Study
Source: Front Med (Lausanne). 2021 May 5;8:630009. doi: 10.3389/fmed.2021.630009 (PMC8131871; doi:10.3389/fmed.2021.630009)
Supplement: Supplementary file 6 [file Table_2.doc]

**Supplementary Table 2**. Echogenicity of the tendons, reference muscles and reference subcutaneous tissue used

for calculating echogenicity ratios of tendon-to-muscle and tendon-to-tissue

|  | **Sarcopenia (+)**  **(shoulder=112)** | **Sarcopenia (-)**  **(shoulder=112)** | ***p* value** |
| --- | --- | --- | --- |
| **Biceps long head tendon** | | | |
| Tendon (pixel) | 103.13 ± 17.05  (99.93 to 106.32) | 105.35 ± 19.14  (101.77 to 108.94) | 0.359 |
| Deltoid muscle (pixel) | 81.08 ± 23.05  (76.76 to 85.40) | 58.88 ± 12.99  (56.45 to 61.32) | <0.001* |
| Subcutaneous tissues (pixel) | 61.65 ± 19.26  (58.04 to 65.26) | 58.07 ± 15.17  (55.23 to 60.91) | 0.124 |
| **Subscapularis tendon** | | | |
| Tendon (pixel) | 74.15 ± 13.84  (71.56 to 76.74) | 77.33 ± 18.28  (73.91 to 80.75) | 0.144 |
| Deltoid muscle (pixel) | 81.89 ± 26.98  (76.84 to 86.95) | 57.12 ± 13.08  (54.67 to 59.57) | <0.001* |
| Subcutaneous tissues (pixel) | 64.17 ± 18.61  (60.69 to 67.66) | 62.36 ± 16.72  (59.23 to 65.49) | 0.445 |
| **Supraspinatus tendon** |  |  |  |
| Tendon (pixel) | 71.95 ± 19.68  (68.26 to 75.63) | 74.80 ± 21.78  (70.72 to 78.88) | 0.305 |
| Deltoid muscle (pixel) | 88.87 ± 27.85  (83.66 to 94.09) | 60.73 ± 14.10  (58.09 to 63.37) | <0.001* |
| Subcutaneous tissues (pixel) | 69.11 ± 20.10  (65.35 to 72.88) | 65.60 ± 15.14  (62.77 to 68.44) | 0.141 |
| **Infraspinatus tendon** |  |  |  |
| Tendon (pixel) | 78.25 ± 20.95  (74.32 to 82.17) | 81.92 ± 18.25  (78.50 to 85.34) | 0.164 |
| Deltoid muscle (pixel) | 91.01 ± 24.24  (86.47 to 95.55) | 70.33 ± 12.39  (68.01 to 72.66) | <0.001* |
| Subcutaneous tissues (pixel) | 64.05 ± 17.21  (60.82 to 67.27) | 64.08 ± 17.03  (60.89 to 67.27) | 0.989 |

Values are given as mean ± standard deviation and 95% confidence interval. * indicates p < 0.05.
